# Design and Evaluation of a Sensor-Instrumented Clutch Mechanism for Quasi-Passive Back Exosuits

**DOI:** 10.1109/TBME.2025.3540625

**Published:** 2025-07

**Authors:** Paul R. Slaughter, Shane T. King, Cameron A. Nurse, Chad C. Ice, Michael Goldfarb, Karl E. Zelik

**Affiliations:** Vanderbilt University, Nashville, TN 37235, USA.; Vanderbilt University, USA.; Vanderbilt University, USA.; Vanderbilt University, USA.; Vanderbilt University, USA.; Vanderbilt University, USA.

**Keywords:** Wearable robots, exoskeletons, wearable sensors, ergonomics

## Abstract

**Objective:**

We designed, built, and evaluated a new sensor-instrumented clutch to expand the capabilities of quasi-passive back exos (exoskeletons and exosuits) to include force sensing, posture sensing, and versatile mode switching. Quasi-passive back exos provide workers with lifting assistance, which can reduce their back injury risk. Central to their design is a clutch mechanism that enables the exo to assist when engaged and be unobstructive when disengaged. However, current exo clutches can have limited sensing and control capabilities.

**Design and Methods:**

We designed a new clutch that integrates an encoder, solenoid, inertial measurement unit, and microprocessor to estimate exo assistance, monitor posture, and switch between engaged and disengaged modes. To validate the new capabilities, 6 participants wore a back exo during stoop and squat tasks. Data from the clutch’s encoder were used to estimate assistance and trunk-thigh flexion angle, then compared to motion analysis lab measurements.

**Results:**

The prototype estimated exo assistance with an average error of 8.8 N (0.9 Nm of lumbar torque) and trunk-thigh angle with an average error of 6.7°. This prototype also maintained the core capabilities of a quasi-passive exo by with-standing 350 N of force when the clutch was engaged, exerting 7–20 N when disengaged, and switching between clutch modes in 0.1 seconds.

**Conclusion:**

We demonstrated an instrumented clutch that enabled exo assistance and posture monitoring, and more versatile control options, in addition to providing back relief.

**Significance:**

This clutch increases the capabilities of quasi-passive back exos, opening new opportunities for exo research and applications.

## I. Introduction

Low back disorders are common and costly, especially among workers who perform repetitive lifting and bending tasks [1], [2]. Lifting and bending contribute to overexertion injury risks [3], [4], [5]. These risks can be mitigated through ergonomic controls, such as eliminating certain material handling tasks. However, it is not always feasible to eliminate risk exposure, and back injuries continue to make up 35% of all workplace musculoskeletal injuries in the U.S. [6].

Exos (including rigid exoskeletons and soft exosuits) are emerging wearable technologies that reduce risks for low back disorders. Back exos have been shown to reduce low back disorder risk factors, like muscle activity [7], spine compression forces [8], and lumbar moments [9]. Recent longitudinal industry studies have also found that back exos reduced back injury incidence amongst warehouse workers [10], [11].

Exos include devices that are powered (motorized), purely passive (elastic), and quasi-passive (elastic with mode switching, such as the ability to toggle assistance on and off). Each class of exo has certain advantages and disadvantages, but these are difficult to generalize because they depend on specific designs and use cases. In this study, we focused on quasi-passive back exos due to their balance between lifting assistance and practical usability. We have been researching and developing these devices in our university lab over the last several years [12], [13], [14] and they are now used in various industries and workplaces around the world [15], [16]. This type of exo provides lifting assistance using a spring (e.g., elastic band) connected between upper body and leg interfaces, and includes a clutch mechanism for adjusting the assistive force from the exo.

Most quasi-passive back exos assist during lifting and bending by using a clutchable spring. This clutch is important because it provides three core capabilities: (1) an engaged mode for lifting assistance, (2) a disengaged mode for unobstructed motion, and (3) a means of switching between engaged and disengaged modes. When the clutch is in engaged mode, the user receives exo assistance during lifting and bending in the form of an extension moment about the lower back. This moment is created by an assistive spring which stretches as a user’s trunk or thighs flex [17], [18]. When the clutch is disengaged, the assistive spring is bypassed, by either decoupling it or configuring it to be in series with a weaker spring, which offers negligible resistance to trunk and thigh flexion. Disengaged mode allows for tasks like sitting or climbing stairs to be performed unimpeded [19]. The mode switch capability allows the user to quickly toggle between engaged and disengaged modes, a feature that distinguishes quasi-passive exos from purely passive exos (i.e., non-mode switching).

Sensors and actuators are not required in quasi-passive exos, but have the potential to enhance the device capabilities [20]. For instance, sensors could measure an exo’s lifting assistance in engaged mode. This added capability could be used to monitor how much assistance is experienced by different users or to quantify the injury risk reduction benefit across different jobs to inform exo implementation [21]. Sensors could also be used to estimate a user’s trunk flexion in disengaged mode. This added capability could monitor a user’s posture to understand how much or how often they are bending. It could also identify their posture when the exo is switched into engaged mode, which impacts the assistance spring’s set point and thus the magnitude of assistance provided by the exo. A variety of sensor signals (e.g., based on a user’s voice, motion, muscle activity, or spatial location) could be used as inputs to algorithms that automatically turn on or off assistance. We refer to this ability to use a variety of different sensor inputs as mode-switching versatility. This versatility might be useful in situations where it is difficult to manually switch modes, such as when both of a user’s hands are occupied. Although we have previously shown proof-of-concept devices using electronic touch sensors, phone apps, and voice activation to mode switch [15], [16], no formal testing or validation was performed with these prototypes. Furthermore, current commercial quasi-passive back exos (e.g., HeroWear Apex 2, Auxivo LiftSuit 2) are engaged/disengaged manually, and do not contain the sensors or actuators needed for the capabilities outlined above.

The objective of this paper is to present the design and evaluation of a new instrumented clutch mechanism for back exos. This clutch includes integrated sensors and actuators that enhance a quasi-passive exo’s core capabilities. In this paper, we focus on the hardware challenges of designing and evaluating the clutch. Software challenges related to control algorithms and intent recognition are beyond the scope of this work, but are briefly discussed in the Discussion. Our high-level design goal was to enable force sensing when the exo was engaged (in assistance mode), enable posture sensing when the exo was disengaged, and increase mode switching versatility so that a variety of control signals could be used to engage and disengage assistance.

## II. Methods

### A. Design Criteria

We created a set of design criteria, which we subdivided into core capabilities, added capabilities, and usability considerations. Core capabilities reflect features or functions in existing quasi-passive exos that we consider crucial and must be retained (Table I). Added capabilities refer to the enhanced features or functions that we sought to incorporate (Table I). Usability considerations were based on user needs and exo adoption barriers identified in prior literature, as well as during our experience developing and testing occupational exos.

We defined several design criteria for our prototype to ensure it retained core capabilities. First, in engaged mode, the clutch and cable must be strong enough to support 350 N, which corresponds with 35 Nm of lumbar torque (assuming 10 cm moment arm [22]). This is within a typical assistance range for current commercial back exos [21], [23]. Second, in disengaged mode, we wanted the user to experience less than 32 N of force on the cable, or less than 3.2 Nm of lumbar torque. This value was chosen to be similar to the disengaged mode resistance we measured in the first HeroWear Apex exosuit, which users found acceptable in terms of user experience and freedom of movement [24]. Third, we desired mode switching in under 1 second. This was based on testing we performed during user-centric development of an exosuit for soldiers [14]. Specifically, we had individuals perform a series of tasks that required switching modes and we found that when mode switching took under 1 second this was acceptable to users.

We then defined design criteria for the added capabilities (Table I). First, in engaged mode, we sought to monitor the force in the exo’s assistance spring as someone bends, denoting the assistance force. It was unknown exactly what measurement accuracy would be required. But for this initial design paper, we targeted an average error of ±25 N which would equate to ±2.5 Nm of extension moment (assuming a 10 cm moment arm between the lumbar spine and the spring [22]). Second, in disengaged mode, we targeted measuring the trunk flexion angle relative to the thigh, denoted trunk-thigh flexion, with an average error of ±5°. It was also unknown what accuracy was needed for this measurement, but we expect that being within about 5° would be as accurate or more accurate than the visual or video inspection procedures commonly used by safety personnel performing risk assessment [25], [26]. Furthermore, 5° is small relative to the coarse posture categorizations (e.g., 20–40°) used in common ergonomic assessment tools in industry such as REBA [27], [28]. Third, to achieve mode-switching versatility, we designed a clutch that could be engaged or disengaged using a common microcontroller output. Our specific design criterion was to demonstrate that a 3.3-volt trigger pulse could engage and disengage the clutch, which would allow a variety of different sensors and algorithms to control the clutch.

In adding these sensing and control capabilities, we also sought to retain the usability of existing quasi-passive exos. We did not define specific or quantitative requirements for usability, but nonetheless these considerations influenced our design thinking. For instance, we wanted to keep the device lightweight and low profile. These considerations were informed by the size and weight of current back exosuits (e.g., HeroWear Apex 2 and SABER, [14]) and our own experiences developing and testing exosuits in work environments. We also preferred to keep the electronics and sensors co-located on the exo for practical and future-looking reasons related to washability, manufacturing, and servicing; factors that become more important at the commercial product stage. Finally, we selected electronic components such that the design could be sufficiently low power to enable a full day of use.

### B. Design Overview

**Fig. 1.**
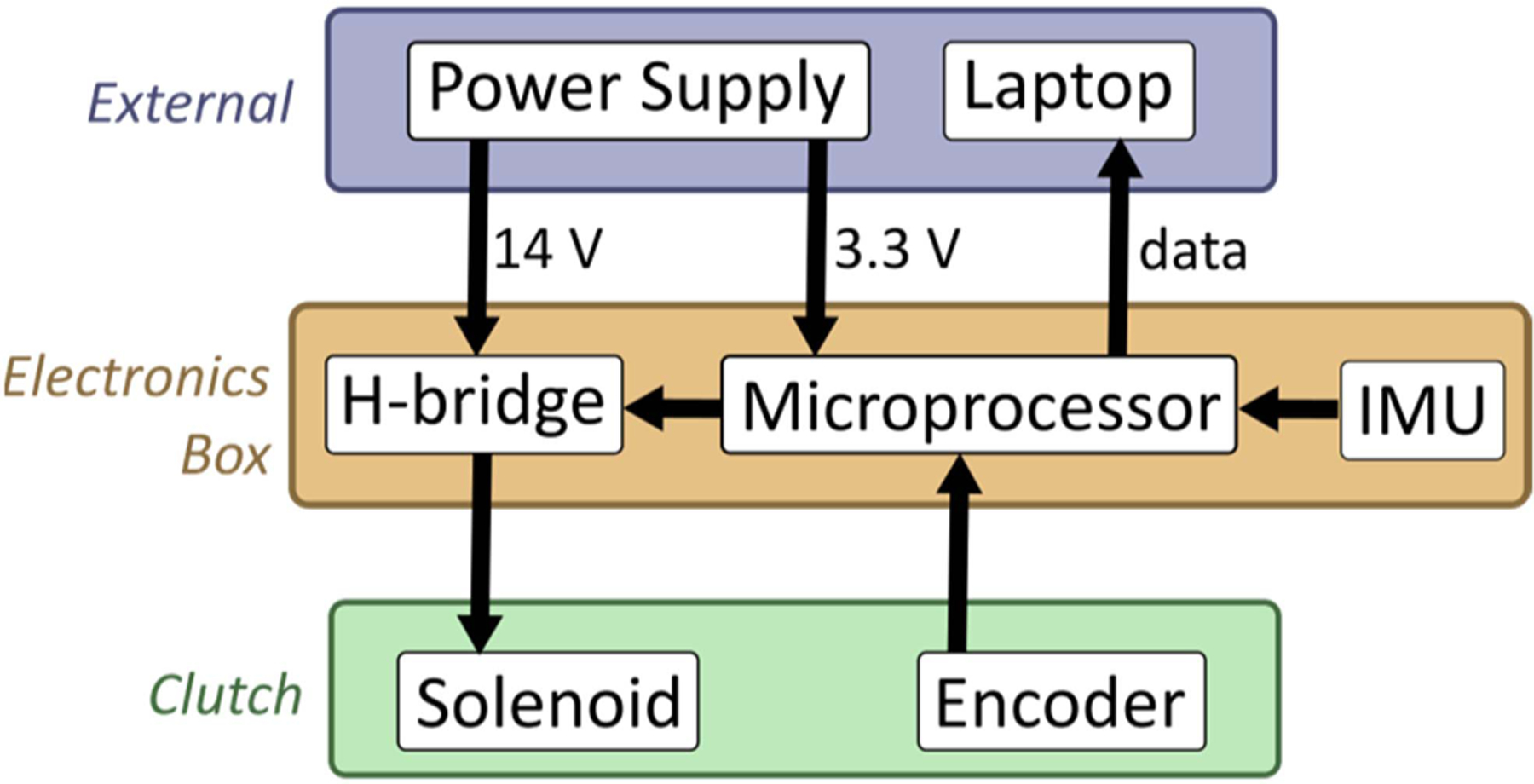
Flowchart of the sensing and electronic components for the instrumented clutch.

We designed a quasi-passive back exosuit. The term exosuit refers to an exo comprised largely of soft materials such as textiles and elastomers. The exosuit is made up of thigh sleeves, elastic bands, and a clutch mounted to a trunk harness. In total, the exosuit prototype we built weighs 1.4 kg and protrudes a maximum of 5 cm from the back. The novel part of the system is the clutch, which is comprised of hardware, sensors, and electronics (Fig. 1). The harness, thigh sleeves, and elastic bands are similar to existing and previously published exosuits [14], [18], [29], and thus are only briefly described.

#### 1) Hardware Design:

**Fig. 2.**
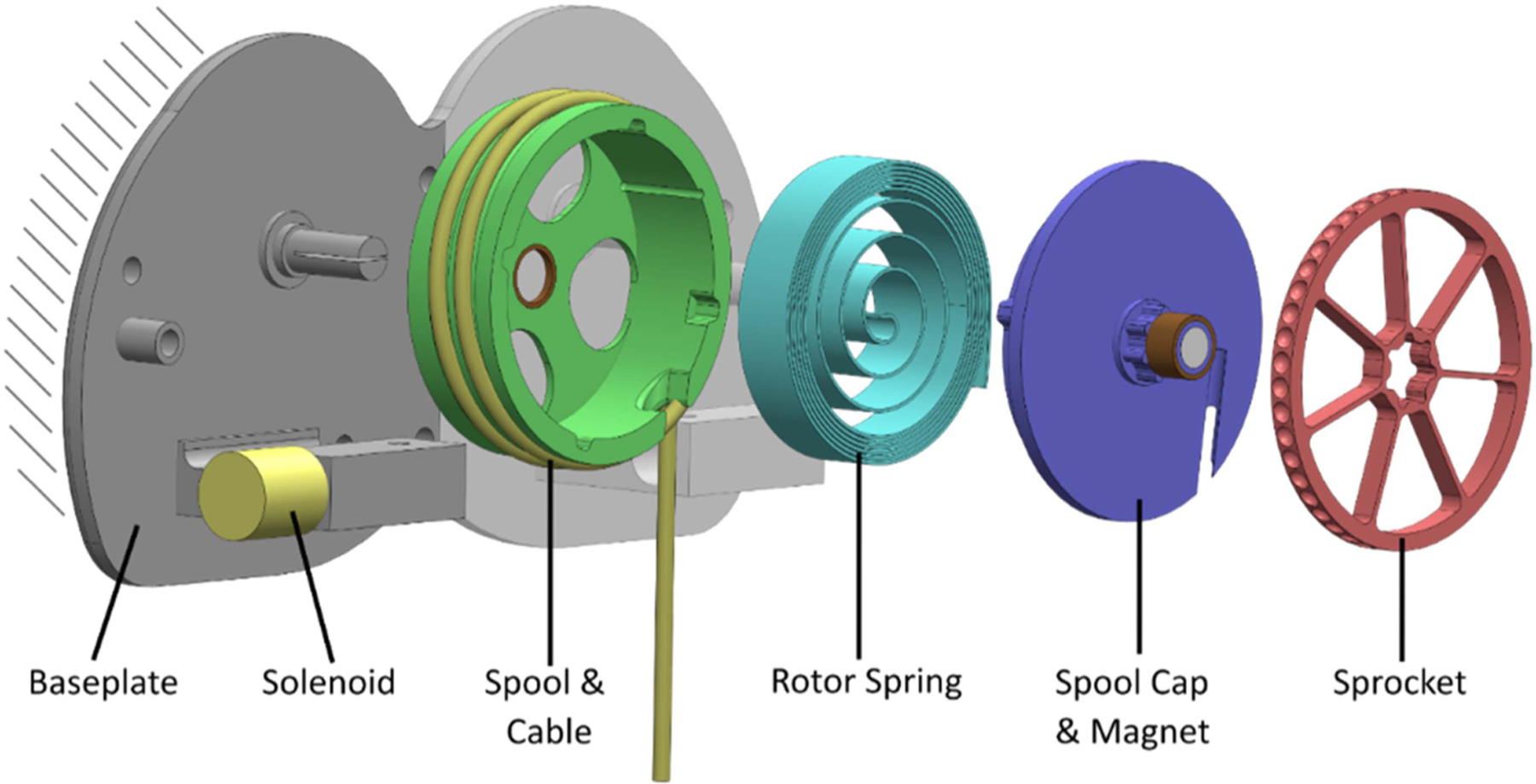
Internal view of the clutch mechanism. Key components are shown, specifically the base plate, solenoid, and spool stack (spool, cable, rotor spring, spool cap, and sprocket). The clutch cover (not shown) fits over the clutch mechanism and positions an encoder over the magnet in the spool cap, so that the encoder can measure the rotation of the magnet as it rotates with the spool.

**Fig. 3.**
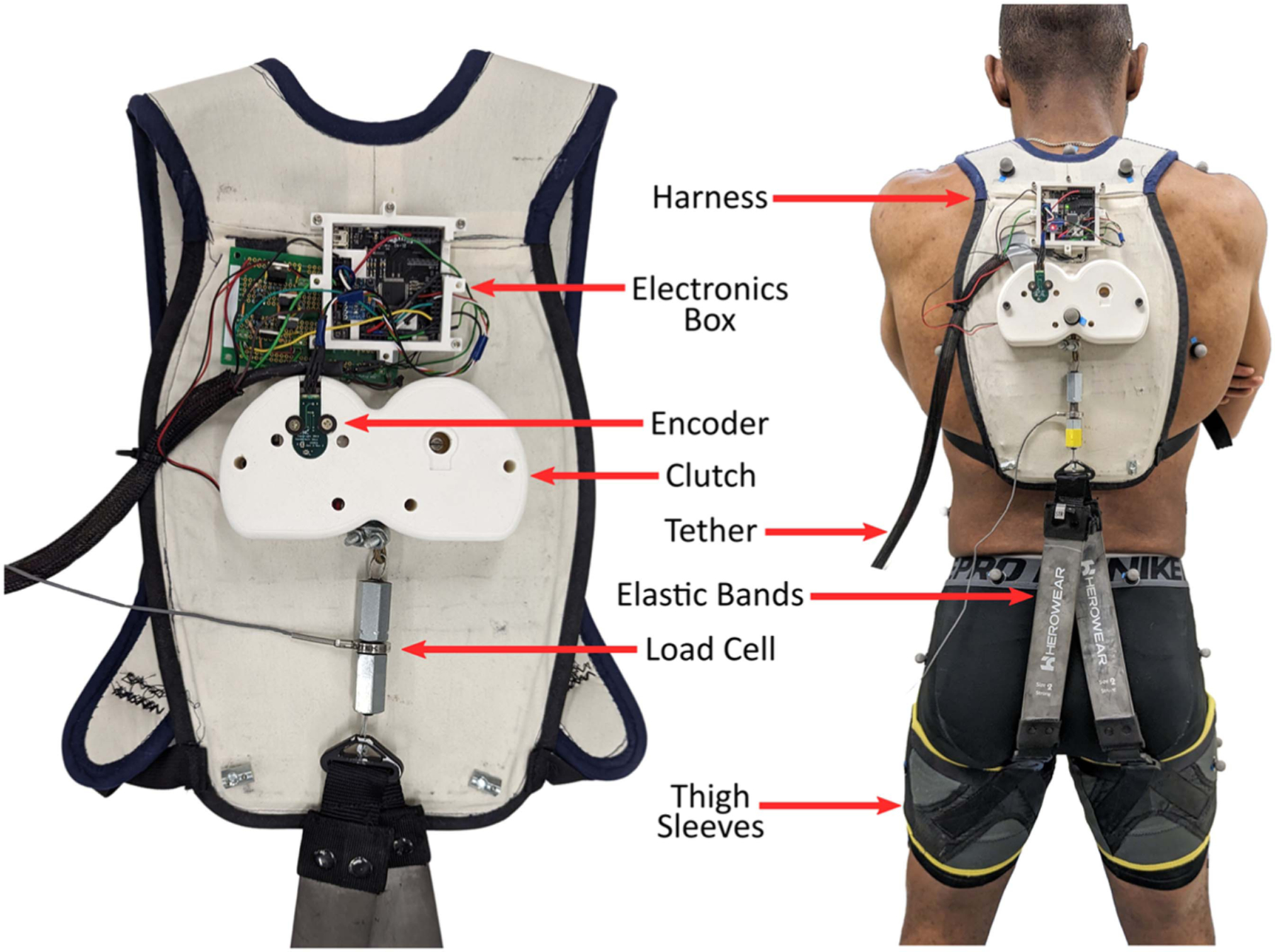
Overview of the instrumented clutch and exosuit. On the left, the clutch and electronics box are mounted to the harness. On the right, this harness is shown on a participant connected to the elastic bands and thigh sleeves. Note that the left prototype has a larger green h-bridge circuit, but on the right, this was miniaturized and placed inside the electronics box.

The clutch mechanism (Fig. 2) has two modes: engaged and disengaged. It is comprised of an aluminum base plate, spool stack (aluminum spool, stainless steel rotor spring, aluminum spool cap, steel sprocket), and solenoid. When the clutch is in disengaged mode, the spool stack can rotate about a center axis that is affixed to the baseplate. This allows a steel cable to be unspooled from the rotating spool stack. A rotor spring is anchored between the center axis and the spool stack such that it provides a low rotational torque (up to 0.7 Nm) that limits the slack in the cable, like a keychain retractor. When in engaged mode, the spool stack is locked in place and cannot rotate, which prevents the cable from unspooling. The cable is in series with elastic bands, which run over the back and buttocks and connect down to the thigh sleeves (Fig. 3). These elastic bands serve as the exosuit’s assistance spring: when the user bends forward, the elastic bands stretch, which creates an assistive extension moment about the lower back and hips.

**Fig. 4.**
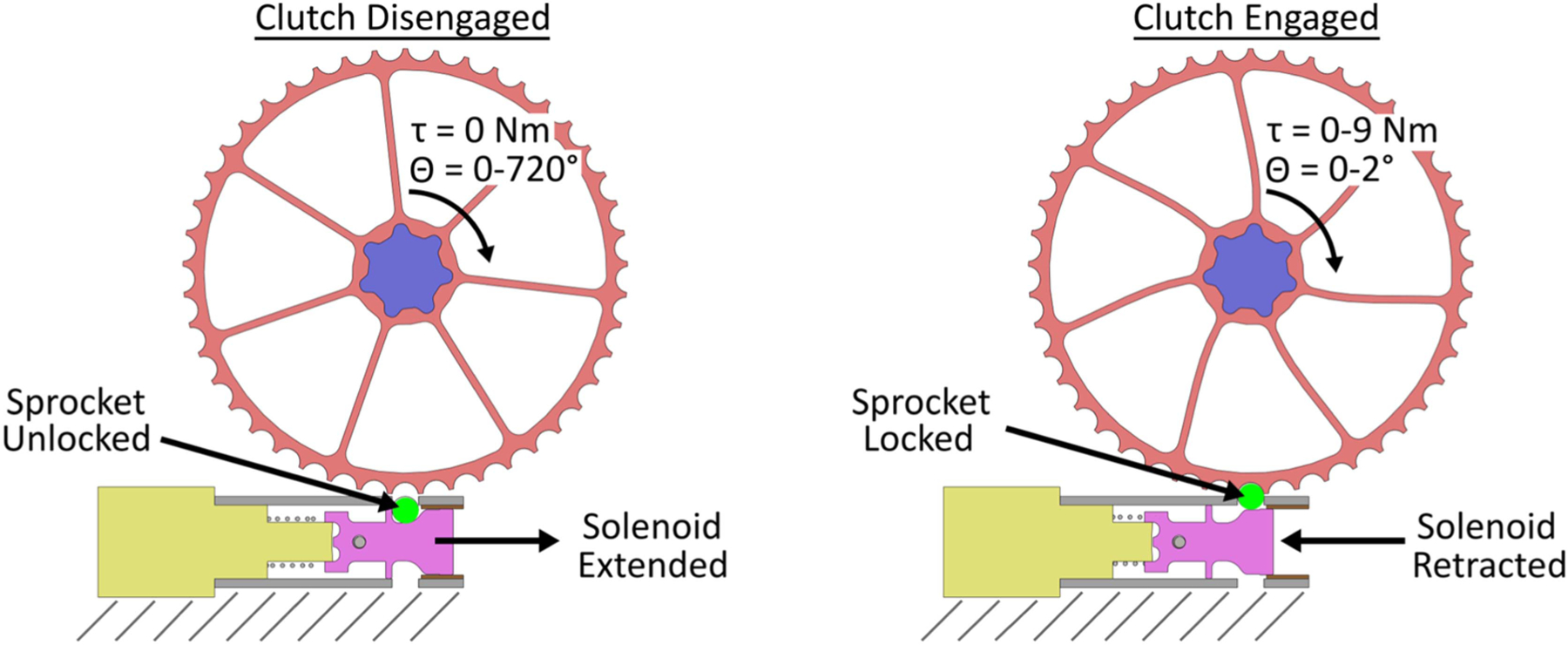
Encoder measurements when the clutch is disengaged (left) and engaged (right). Left: The sprocket’s outer rim is unlocked and can rotate as the user moves. This rotation increases with trunk or thigh flexion and is measured by the encoder. Right: The sprocket’s outer rim is locked by the ball bearing (green circle). Loading the cable creates a torque around the center axis, which deflects the spokes of the sprocket. This rotational deflection increases with torque magnitude and is measured by the encoder. The torque (*τ* ) and angle (*θ*) values depicted in the figure provide approximate ranges when the exosuit is disengaged (larger rotations, lower loading) and engaged (smaller rotation, higher loading).

The clutch switches modes using a pull magnetic latching solenoid (STA Solenoids ^1^*/*_2_ × ^1^*/*_2_ #231). The solenoid is mounted to the base plate. To engage the clutch, the solenoid retracts a plunger, which pushes a hardened steel ball bearing into a detent on the edge of the sprocket, locking its rotation (Fig. 4). There are 45 detents, allowing the spool stack to be locked every 8°. To disengage the clutch, the solenoid extends. Gravity or any small torque on the spool stack removes the ball bearing from the detent, allowing the sprocket to rotate.

The base plate of the clutch is attached to the harness (Fig. 3), which is worn like a backpack. The diameter of the clutch spool is 5.1 cm and can wind up a maximum of 35 cm of cable. This cable is then attached to off the shelf elastic bands (S1500 Size 2) and thigh sleeves (L/XL) from a commercial exosuit (HeroWear Apex). The clutch mechanism (Fig. 2) has a footprint of 15.2 cm by 8 cm and protrudes from the harness by 4 cm. It weighs 450 grams and has the capacity for two identical (mirrored) clutch mechanisms, which would enable independent control of the left and right elastic bands, or of two parallel sets of bands. For this prototype and evaluation, only one spool stack was populated so that the left and right elastic bands were controlled together.

#### 2) Sensing and Electronics:

A single encoder along the center axis of the spool stack is configured to add two capabilities to the clutch: estimate the user’s posture (trunk-thigh flexion angle) in disengaged mode and estimate exosuit assistance force in engaged mode. Sensing is provided by a high-resolution incremental quadrature encoder (AMS AS5145B) located on the clutch cover, which measures the rotation of the magnet in the spool cap as it rotates with the spool. When the clutch is disengaged, the encoder measures the spool’s rotation, which increases with trunk and hip flexion (Fig. 4, Left). When the clutch is in engaged mode, the rotation of the outer rim of the spool is locked. However, the spokes of the detent sprocket act as a series elastic element which deflects rotationally and in proportion to the force exerted by the cable and elastic bands (Fig. 4, Right). The encoder measures this spoke deflection as rotation of the spool about the center axis, allowing the exosuit’s assistive force to be estimated.

Additional sensing is provided by an inertial measurement unit (IMU, Adafruit MPU 6050), which is located in the electronics box on the upper back. The IMU measures 6-axis acceleration and gyroscope signals which can be used to estimate trunk orientation (e.g., using a complementary or Kalman filter). This IMU can also be used to control the mode of the clutch. To demonstrate control proof-of-concept, a vertical acceleration threshold of 5 m/s^2^ was used to command a mode switch, either from engaged to disengaged mode or disengaged to engaged mode. As a simple demonstration, we had one participant perform calf raises which causes a clear spike in vertical acceleration. We then confirmed that this successfully switched clutch modes without the need to manually trigger.

We configured a microprocessor (Microchip dsPIC-33FJ32GS608) to control the solenoid and export sensor data. The microprocessor controls a custom H-bridge with a 3.3-volt output, which subsequently powers the solenoid with ±14 volts at 1 amp. The latching solenoid helps limit power consumption by only requiring power to switch modes (*<<*1 second), not when maintaining either mode. Additionally, the microprocessor exports rotation data from the encoder and 6-axis acceleration and angular velocity data from the IMU. For this proof-of-concept prototype, the microprocessor and solenoid were powered with an offboard power supply and all sensor data were exported via tether to a laptop for data logging. For a fully portable version, batteries and data storage could be added to the electronics box.

As part of our design process, we confirmed that a small battery would be sufficient to power a portable version of this exosuit. To do this, we measured power consumption from the prototype during typical use and assumed mode-switching once per minute for 8 hours (which is a very high-end estimate of mode-switching we expect for most use cases). This amounts to a baseline power of 0.80 W, a maximum power of 14.8 W during mode switching, and a required battery capacity of 2070 mAh. Assuming we used a small battery like a Turnigy nano-tech 4S 50C (14.8 V, 2200 mAh, 100×35×30 mm), we could power the exosuit (i.e., clutch actuation, sensors, microprocessor) throughout a full workday while adding just 218 g to the total weight.

### C. Design Evaluation

We validated the core and added exosuit capabilities through benchtop testing and human-subject testing.

#### 1) Benchtop Testing:

We used a force gauge (Extech 475044) to measure the retraction force on the cable when the clutch was disengaged. We recorded a force reading in increments of 1 cm from 1 to 35 cm of extended cable (maximum extension).

We used a video camera (GoPro 8) to record and time clutch mode switches. We triggered 20 consecutive mode switches by doing calf raises. When the mode switch was detected via the IMU data, an onboard LED would switch on, and the solenoid would receive power for 1 second. We measured the time it took for the clutch to switch modes by counting the number of video frames between the light turning on and the solenoid plunger being fully extended (for disengaged mode) or retracted (in disengaged mode). We performed 10 disengaged switches and 10 engaged switches and calculated the average switching time.

We hung a 36 kg plate mass (corresponding to 353 N of force, or about 35 Nm of torque about the low back, per our design requirement) from the cable with the clutch mounted vertically. The mass was manually placed on and off the cable 10 times. Afterwards, we visually inspected the clutch mechanism for any hardware damage or failures.

#### 2) Human Subject Testing:

We tested the clutch capabilities on 6 healthy individuals (3 males, 3 females, average age ± SD = 26.0 ± 2.3 years, average height ± SD = 178 ± 6 cm) who provided written informed consent to participate in this study which was approved by Vanderbilt University’s Institutional Review Board (#141697). We helped participants don the exosuit, then participants performed bending motions as data were collected from the clutch’s sensors and lab-based instrumentation.

Motion capture and clutch data were synchronously recorded. To track segmental position, motion capture markers (VICON) were placed on the thighs, pelvis, and trunk using standard marker layouts (Thigh 1, Pelvis 4, and the Thorax 1, [30]. For the disengaged mode trials, a load cell (FUTEK LCM200) was placed in series between the clutch cable and the elastic bands. Data from the clutch (6-axis data from the IMU, rotation data from the encoder, and the sync signal) were simultaneously recorded to a laptop (MATLAB Simulink 2021b) via the tether. Clutch data and load cell data were collected at 1000 Hz and then downsampled to 200 Hz to match the 200 Hz motion capture data. Participants performed 90 bending motions, half in engaged mode and half in disengaged mode, while data from the clutch and lab instrumentation were collected. The same 45 bending motions were performed in both engaged and disengaged modes. The 45 bending motions were divided into 3 different data collection trials consisting of static stoops, dynamic stoops, and squats. For the static stoop trials, participants were instructed to perform a stoop (maintaining extended legs and flexing their trunk through hip flexion) but to hold the bent posture for 2 seconds before standing back up. For the dynamic stoop trials, participants were instructed to perform a stoop, but without pausing for two seconds. For the squat trials, participants were instructed to dynamically perform a squat (flexing their trunk through a combination of knee and hip flexion), without pausing at the lowest point. For each of these 3 motions, 15 bends were performed. Participants were instructed to perform the first 5 trials as deep bends, the middle 5 as moderate bends, and the last 5 as shallow bends. These depths were chosen to represent a broad range within each type of bending (stooping or squatting) motion. The clutch was engaged when each participant was standing upright, and disengaged after performing the first set of 45 bending motions.

#### 3) Data Processing:

In summary, we evaluated whether the encoder data could be used to estimate the exosuit force and trunk-thigh flexion angle for each participant’s bending motions. In custom MATLAB code, we created testing datasets to establish linear regression models between the encoder data and the ground truth (lab-based) measures of force and angle. We then applied these models to independent testing datasets and measured the errors between these estimated forces and angles, and the corresponding ground truth data. We created models for when the clutch was engaged (and estimating assistance force) and disengaged (and estimating trunk-thigh angle). We first created models using a subject-specific training dataset and then second created models using a subject-independent training dataset to better understand the generalizability of this approach.

We processed the encoder data to remove non-bending data (standing between trials). This involved the following steps: zeroing the encoder when the participant was standing upright, using negative to positive sign changes in trunk-thigh angular velocity to identify the start and end of each bend, and then removing all of the data in-between bend end/start frames and any encoder data that was not greater than 0 to isolate the bending data from the standing data. The encoder data from the engaged mode trials had to undergo further data processing to account for direction-change hysteresis built into the encoder sensor we used. To account for this hysteresis, we performed two operations: (i) we subtracted 0.5 degrees from encoder signals that were decreasing, representing when the elastic bands were being unloaded during trunk extension, and (ii) we removed encoder datapoints within the hysteresis dead band that occurs just after the exosuit force peaks or reaches zero.

For each participant, we created 2 subject-specific models—one for estimating assistance force when the clutch is engaged and one for estimating trunk-thigh angle when the clutch is disengaged. For both models, data from the first deep static stoop, the first deep dynamic stoop, and the first deep squat were allocated to a training dataset, with the other 42 bends being the testing dataset. The three bend trials used in the training set were chosen to reflect the motions performed, while also being small enough to not overfit the model to the data. For the engaged mode training dataset, a 1st order polynomial fit with least absolute residuals was applied to create a linear model (relationship) between the encoder data and the ground truth assistance force. The same type of fit was applied to the disengaged mode training dataset to create a linear model between the encoder data and the ground truth trunk-thigh angle. We also created 2 subject-independent models for engaged and disengaged modes using leave-one-out cross validation. For these subject-independent models, the training dataset was 5 subjects, with the remaining subject’s data representing the testing dataset. Then, we created the same types of linear fits as in the subject-specific models to estimate force and angle from the encoder data.

Here we briefly expound upon the rationale for exploring subject-specific and subject-independent models. These subject-independent models could be applied to anyone without needing unique calibration, but potentially at the expense of accuracy since it assumes all users are the same. In contrast, the subject-specific models (detailed above) were created because differences in body shape, body size, and bending form may impact the relationship between the spool rotation measured by the encoder and the relevant biomechanical angles and forces. However, this would require the model to be calibrated to each user.

These models (subject-specific force and angle, subject-independent force and angle) were applied to the encoder data in each respective testing dataset to estimate assistance forces and trunk-thigh angles. We filtered the load cell data using a bidirectional 15 Hz Butterworth filter and considered this the ground truth assistance force. Similarly, we computed the ground truth trunk-thigh flexion in C-Motion’s Visual3D using the sagittal angle between the left thigh and trunk segments after applying a bidirectional 8 Hz Butterworth filter. We compared the estimated forces and angles to their respective ground truths and calculated a mean absolute error (MAE) and coefficient of determination (R^2^) for each subject across their time-series bending data. We also calculated MAE for two discrete values, the maximum bending angle and maximum assistive force for each bend in disengaged and engaged mode, respectively.

## III. Results

The benchtop evaluation confirmed that this new clutch design satisfied the core exo capabilities (Table II). In engaged mode, the clutch design withstood our max force goal of 350 N during the benchtop test without any visual indications of damage. This would equate to 35 Nm of extension moment about the low back assuming a 10 cm moment arm between the elastic bands and the L5-S1 joint. Of note, in human subject testing, we measured up to 270 N (27 Nm) of assistance during stooping and squatting. In disengaged mode, the cable tension was measured to be 7 to 20 N when the cable was unspooled from 1 cm to 35 cm, which is less than the 32 N design goal. When mode switching, the average time to disengage the solenoid was 0.05 seconds (range of .01–.14 seconds) and the average time to engage the solenoid was 0.10 seconds (range of .03–.34 seconds); times that were within our 1-second target.

**Fig. 5.**
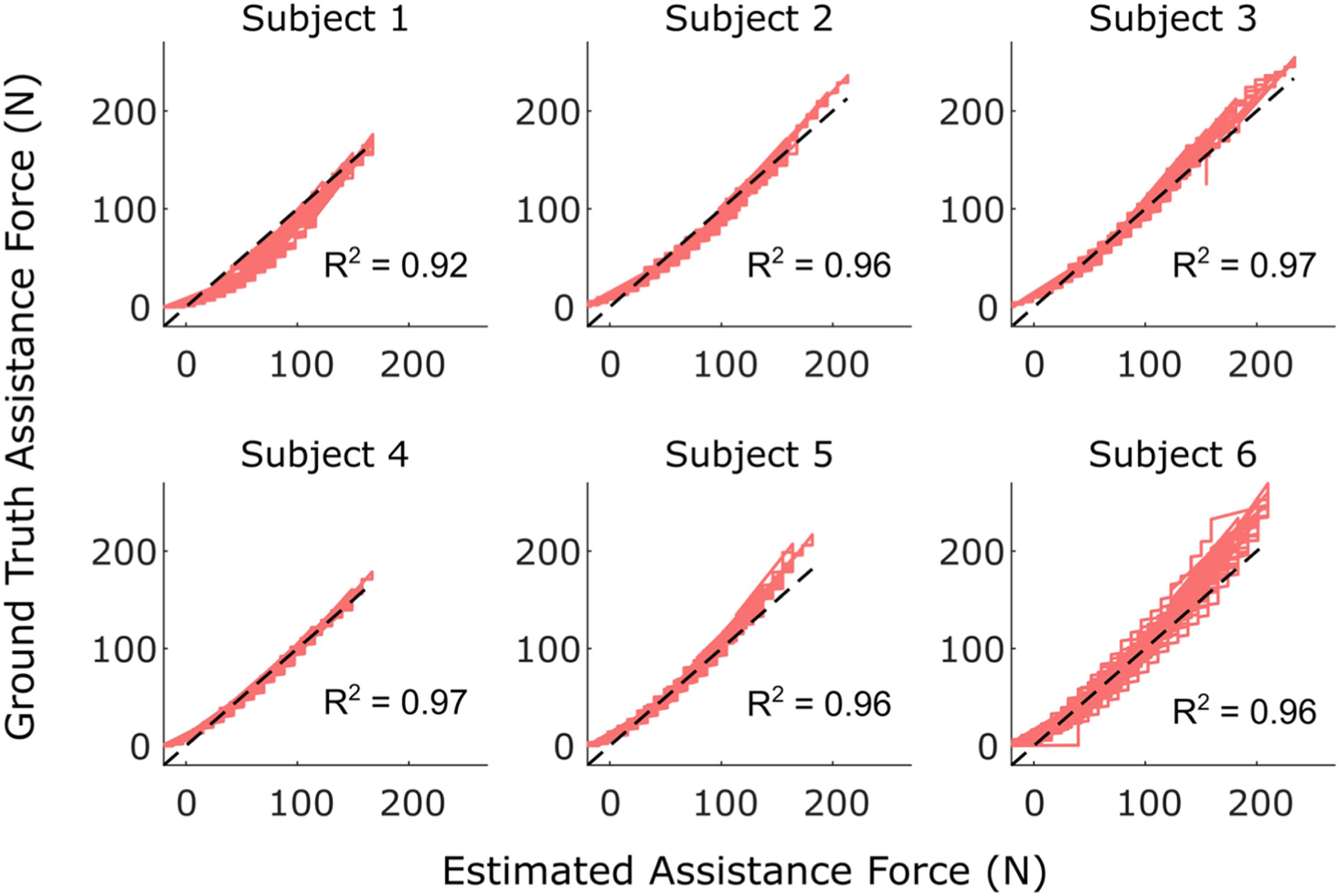
The estimated assistance force is correlated to the ground truth assistance force during all bending trials (stoops and squats) with the clutch engaged. The results shown used the subject-independent model. The black dashed line is a unity line that represents a perfect match between estimated and ground truth.

**Fig. 6.**
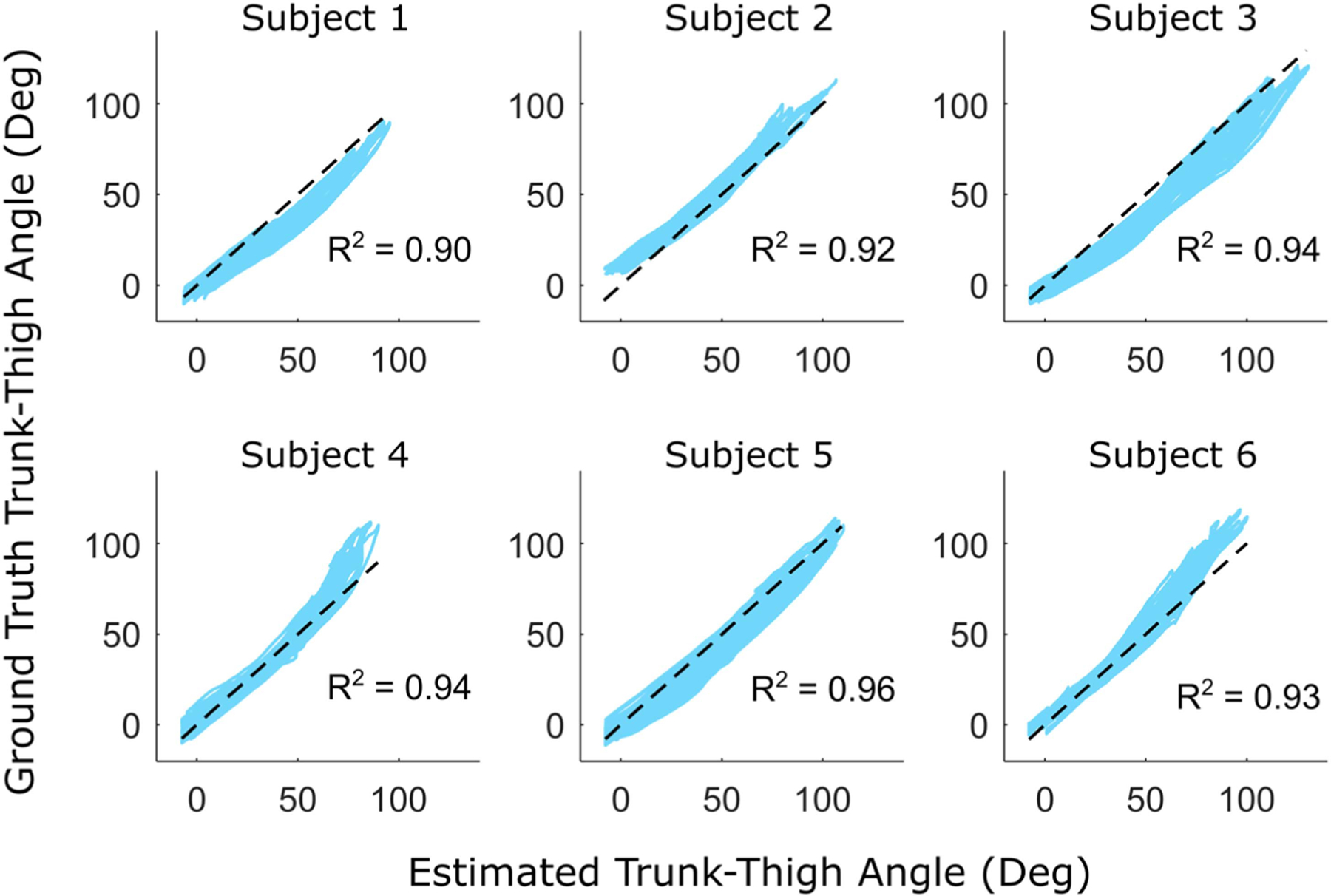
The estimated trunk-thigh angle is correlated to the ground truth angle during all bending trials (stoops and squats) with the clutch disengaged. The results shown used the subject-independent model. The black dashed line is a unity line that represents a perfect match between estimated and ground truth.

The human subject testing confirmed that this clutch design successfully achieved the added exo capabilities. Table III summarizes the accuracy of our estimated force and angle metrics, reporting the range and average MAE across the 6 participants. In engaged mode, the subject-specific and subject-independent models were both able to estimate the assistance force (time-series and peak) within the design goal of ±25 N when the clutch was engaged (Fig. 5) with average subject errors ranging from 7.8 to 12.5 N (Table III). In disengaged mode, the subject-specific model estimated trunk-thigh angle within the design goal of ±5° (Fig. 6) with mean absolute errors of 4.6° for time series and 3.7° peak angle (Table III). The subject-independent model yielded mean absolute errors 6.7° and 8.8° for time series and peak angle, respectively, which was outside of our 5° design goal. These results were averaged across each participant performing both stoops (mostly trunk rotation) and squats (mostly thigh rotation). Participants had an average trunk flexion range of 82°, 68°, and 53° for deep, moderate, and shallow stoops, compared to only 21°, 17°, and 13° of thigh flexion range. In contrast, deep, moderate, and shallow squats had average trunk flexion ranges of just 39°, 33°, and 25° and thigh flexion ranges of 70°, 60°, and 48°, respectively. Mode switching was controlled via a 3.3-volt TTL pulse sent by the microprocessor based on IMU data input, which demonstrated the versatility of control.

## IV. Discussion

We designed a new instrumented clutch and demonstrated that it adds new capabilities to a quasi-passive back exosuit while maintaining the core capabilities and important usability features (Table II). Core capabilities included lifting assistance in engaged mode, transparency in disengaged mode, and quick mode switching. We then incorporated an encoder, solenoid, IMU, and microprocessor into the exosuit clutch to achieve our objective of force sensing, posture sensing, and versatile mode switching control.

The performance of the prototype generally met or exceeded our design and usability targets. For force and posture sensing, we expect that the linear subject-independent models will be sufficiently accurate for estimating time series and peaks in most applications. However, additional accuracy could be achieved by using subject-specific or more complex (non-linear) models. The prototype had a similar weight and form factor to other commercial back exos, which bodes well for usability. We were also able to keep all the sensors and electronics co-located on the rear side of the harness. The prototype offers versatility in how the clutch can be controlled, and power consumption was low enough that a small battery could power it for a full work day (see II.B.2 for details).

**Fig. 7.**
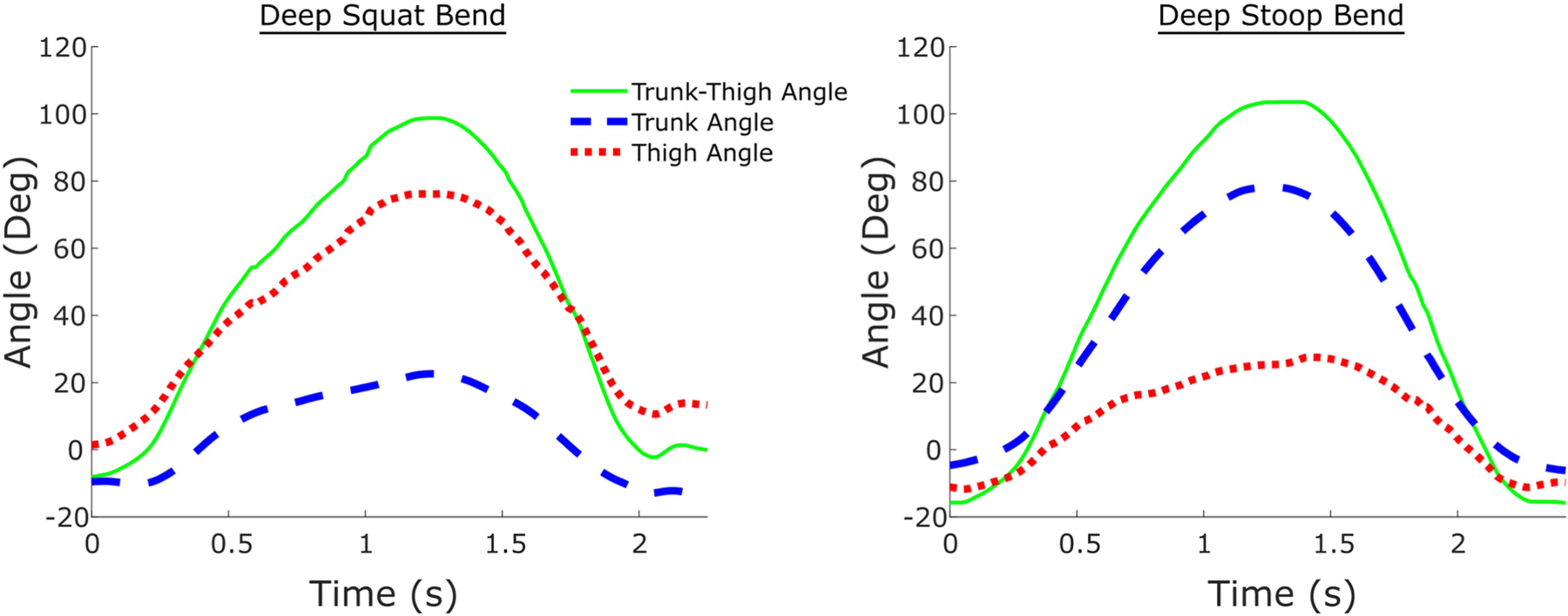
Difference in body flexion angles estimated during a deep squat (left) vs. during a deep stoop (right). Representative results are shown from one participant (subject 5). The difference in thigh and trunk angles can be used to determine lifting posture (squat vs. stoop). The plot on the left shows higher thigh flexion and lower trunk flexion, which indicates that the lift was a squat. In contrast, the plot on the right has higher trunk flexion and less thigh flexion, indicative of a stooping bend. Trunk angles were calculated using the trunk IMU and MATLAB’s *imufilter* function. Thigh angles were calculated by subtracting trunk angle from the trunk-thigh angle estimated with the encoder.

This exosuit provides lifting assistance to a user while tracking assistance and posture, which opens new learning opportunities during field use. Measuring lifting assistance could be used to evaluate which lifts or jobs receive the most offloading benefit from the exo, or paired with existing ergonomic tools to assess the impact of the exosuit on injury risks. This capability could potentially be useful to safety practitioners or researchers performing field studies on exos. The posture sensing capabilities of the clutch could be used to evaluate the number of bends performed, or to understand the distribution of squat vs. stoop bends (Fig. 7) over the course of a user’s work day. The mode switching control signal could also be stored and used in conjunction with posture and force sensing to understand behavioral aspects of exosuit use, such as how often users are engaging/disengaging assistance, or whether certain types of jobs or bends are performed without the clutch engaged.

This type of quasi-passive back exo with an instrumented clutch may serve a unique niche that is not currently filled by existing devices. Commercially, standalone workplace wearable sensors (e.g., StrongArm, MotionMiners, LifeBooster) can be used to monitor body posture; however, these do not measure exo assistance. One exo manufacturer (ErgoSante) has created an add-on sensor to estimate trunk bending and exo assistance; however, it only works for one specific product that uses flexible rods instead of elastic bands. It also does not provide mode switching versatility. Finally, powered exos (e.g., German Bionic CrayX, Verve SafeLift) provide lifting assistance while also monitoring posture and assistance; however, these powered exos are much heavier, bulkier, and costlier than quasi-passive exos due, in part, to the much larger motors and batteries and more complex control required. The clutch presented here uses similar types of mechanisms and sensors as other exoskeleton clutches published in academic studies, which have been used to assist a variety of joints [31], [32]. Mechanically, our clutch locks and unlocks using a latch with multiple locking positions operated by a solenoid, similar to [33]. The encoder also estimates force and joint angles similar to [34], [35], [36], [37]. Some of these designs offer greater accuracy in measuring force or posture, for instance, by adding a dedicated load sensor. However, this is often at the cost of added bulk, weight, or electronics requirements. The optimal balance of clutch features (e.g., sensing range and accuracy) and characteristics (e.g., weight and strength) often depends on the specific user and application, making it difficult to make broad statements about pros and cons of our design relative to other devices. However, unique aspects of our design include using a single encoder to estimate both assistance force and posture, and applying this instrumented clutch to an established and usable back exo design [14], [24]. Overall, our prototype uniquely blends the weight and form factor of quasi-passive back exos with the sensing capabilities of powered exos and wearable sensor systems.

While this study demonstrated proof of concept, there are several limitations and opportunities for future design improvements. We developed and tested a tethered prototype, but data logging and power would need to be added into the electronics box for a fully portable exosuit. We could also automate the post-processing using the on-board controller to allow real-time estimation across postures and clutch locking positions. This prototype was tested under a 350 N static load to confirm function, but in future prototypes cyclic load testing should also be performed to ensure durability. There are also variations of this clutch design that could be further explored. For instance, force sensing could be achieved with other mechanical configurations or by adding a load cell; however, many of these variations are less desirable in terms of weight, form factor, or power consumption. The accuracy of assistance force and trunk-thigh flexion estimates seemed adequate, but further sensing accuracy could be achieved by using an encoder with no direction change hysteresis or by creating a clutch with more compliant spokes [34]. Another important improvement would be making all of the electronics separable from the textile portion of the exosuit to better facilitate cleaning, an important factor for exo adoption [38]. This task is simplified given the centralized location of all of the electronics on the harness (Fig. 3). Regardless of the precise design, a drawback to this concept is the need for this exo to be recharged regularly, which complicates its use relative to existing quasi-passive back exos. Open questions remain related to where and when this instrumented clutch concept may provide sufficient value to offset its added cost and complexity relative to back exos using manually-triggered clutches, or other ergonomic solutions.

In the future, the force and posture estimation models could be further tested and validated using data collected on a wider range of participants and lifting scenarios. This could involve, for instance, asymmetric lifts that include lateral bending or rotation of the trunk. Testing more participants would also help evaluate the generalizability of the estimation models across a variety of user sizes and shapes. Testing a wider range of participant heights would be especially important, since the range of participants tested in this study (168 – 185 cm) may have led to lower angle estimation error. However, more advanced models that adjust for a user’s height could also be developed which we expect could be trained to be at least as accurate as the subject-independent models presented in this study (within ∼5–10°). Further models could also be developed to estimate thigh flexion using the trunk flexion estimate from the IMU and the trunk-thigh flexion estimate from the encoder (example shown in Fig. 7). Separately monitoring trunk and thigh angles could be helpful in identifying higher-risk bending postures and differentiating between squat bends (larger thigh flexion) and stoop bends (larger trunk flexion).

Controlling the clutch electronically expands the device’s potential capabilities, but developing a robust controller poses a significant challenge. A calf raise was chosen to demonstrate this prototype’s capabilities, but mode switching could also be triggered using any other motions or different types of user input (e.g., touch sensor, voice command). A future challenge is developing reliable and practical control methods for specific users or applications. Mode switching could also be performed automatically by predicting when a user may want or not want lifting assistance. However, developing an intuitive controller that is able to respond reliably and quickly is a longstanding challenge in the exo field [39], [40] and requires a deep understanding of an application’s specific users and tasks. This understanding could be informed, in part, by using the recorded sensor signals from this instrumented clutch to learn how and when users prefer to switch modes with the exosuit. Future work is needed to explore and evaluate control algorithms and user intent recognition—with this prototype and other types of electronically-controlled occupational exos.

## V. Conclusion

We presented and evaluated a novel clutch prototype that includes sensors and actuators to expand the capabilities of a quasi-passive back exo. We demonstrated the ability of this design to maintain the core capabilities of a quasi-passive back exo while adding capabilities related to posture monitoring, assistive force monitoring, and versatile mode switching. Future work is needed to develop fully portable hardware and robust control algorithms, and ultimately to explore the utility and trade-offs of this concept relative to other types of exos and ergonomic solutions.

## Figures and Tables

**TABLE I:** Overview of Core Capabilities of a Quasi-Passive Back Exo, and the Added Capabilities We Sought to Incorporate Through the Design of a New Sensor-Instrumented and Actuated Clutch

	Core Capability	Added Capability
**Engaged Mode**	Assist the user’s back during lifting and bending	Measure the level of assistance
**Disengaged Mode**	Allow unobstructed trunk and hip flexion	Measure trunk-thigh flexion angle
**Mode Switching**	Quickly switch between engaged and disengaged modes (manually)	Increase the versatility of mode switching control beyond manual triggering

**TABLE II:** Summary of Target Criteria and Achieved Capabilities for the Clutch

Capability	Criteria	Achieved
Support high lifting assistance forces	350N	350 N
Exert low forces when disengaged	<35 N	7–20 N
Quick mode switching	< 1 sec	0.05–0.1 sec
Estimate assistance	Within 25 N	7.8–12.5 N
Estimate posture	Within 5°	3.7–8.8°
Versatile mode switching	3.3 V Pulse	3.3 V Pulse

**TABLE III:** The Average and Range of Participant’s MAE for Estimated Lifting Assistance and Posture

	Estimating Assistance Force		Estimating Trunk-Thigh Angle	
	Time Series	Peak Force	Times Series	Peak Angle
Subject-Specific Model Error	8.0 N (5.5 N – 10.2 N)	7.8 N (4.5 N – 16.3 N)	4.6° (3.4° – 5.7°)	3.7° (2.7° – 5.7°)
Subject-Independent Model Error	8.8 N (5.6 N – 12.1 N)	12.5 N (4.5 N – 27.6 N)	6.7° (5.6° – 7.2°)	8.8° (4.2° – 16.01
